# Supplementary material for: Neutron-upscattering enhancement of the triple-alpha process
Source: Nat Commun. 2022 Apr 20;13:2151. doi: 10.1038/s41467-022-29848-7 (PMC9021293; doi:10.1038/s41467-022-29848-7)
Supplement: Supplementary file 1 — Supplementary Information [file 41467_2022_29848_MOESM1_ESM.pdf]

Supplementary Information for Neutron-upscattering enhancement of the triple-alpha process

1 Supplementary Note 1

Supplementary Fig. 1 shows an example $^{12}\mathrm{C}(n, n_2)3\alpha$ event. Supplementary Fig. 2 illustrates how the RANSAC algorithm is used to fit 3α-particle events. The next two supplementary figures (Supplementary Figs. 3 and 4) illustrate how the desired inelastic channel is isolated from those that α decay to excited states in $^{9}\mathrm{Be}^{\star}$. The last four supplementary figures (Supplementary Figs. 5-8) show the results of the multi-channel R-matrix fit. Finally, a supplementary table is provided with the states of interest for the R-matrix fits and the resulting parameters.

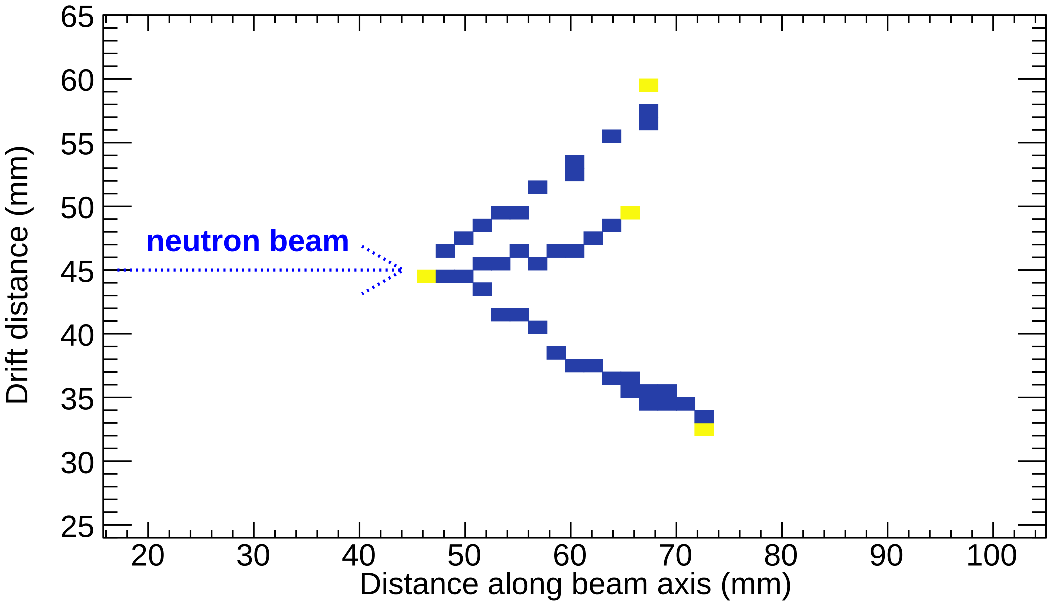

Supplementary Figure 1: An example $^{12}\mathrm{C}(n, n_2)3\alpha$ event track reconstructed within the TPC shown in 2D. The neutron beam direction (dotted blue line) is to the right. The automatically-identified vertex location and three α-particle arms are identified by hot yellow pixels.

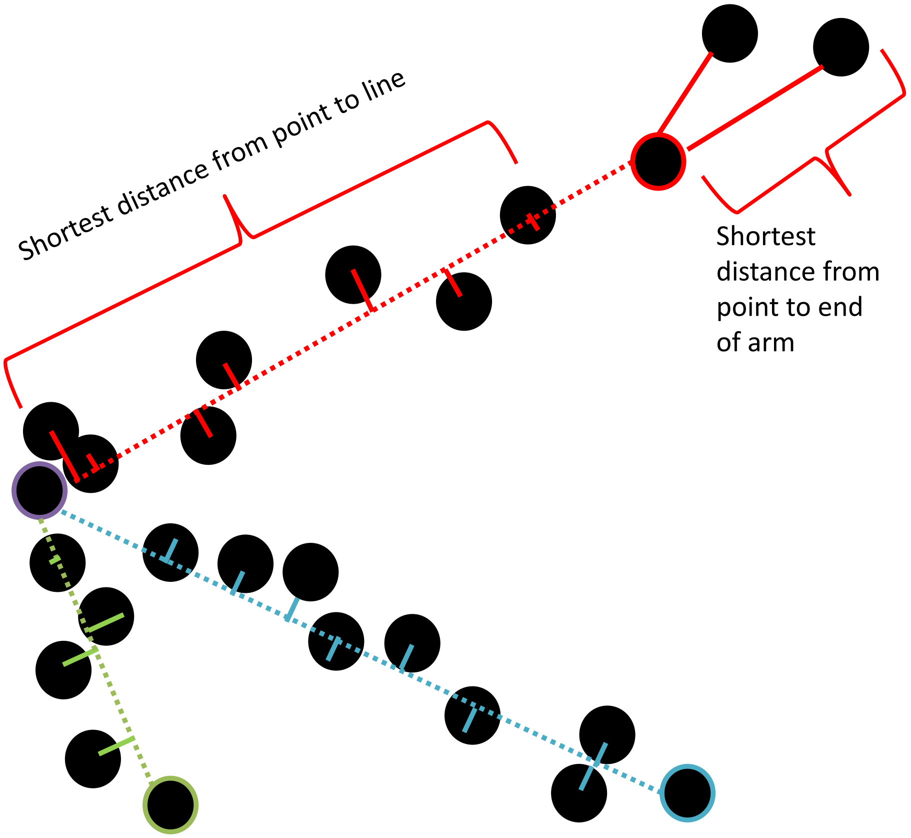

Supplementary Figure 2: Schematic showing the methodology of the RANSAC fitting routine. Three randomly-selected points for each arm (red, blue, green) for each iteration are made and 3D line segments are made between these three points and the vertex (purple). For each point in the 3D point cloud, the perpendicular distance to each of the three lines is calculated. For events where the distance between the point and the decay vertex is larger than the distance between the decay vertex and the arm (e.g. the final two points for the red arm), the 3D distance between the point and the arm point is used instead. The shortest of these three distances for each point is found and summed to provide a total distance-squared for each iteration. The random sample with the smallest total distance-squared is chosen as the best fit. This naturally finds not only the direction of the arms, but selects points at the end of the arm without over-biasing.

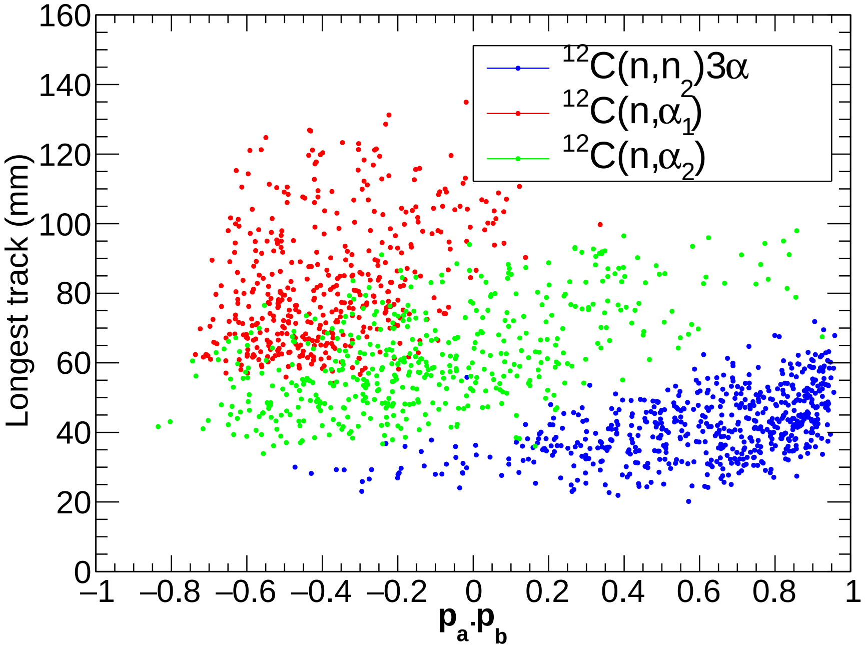

Supplementary Figure 3: Channel separation plot. Simulated data for $E_n = 10$ MeV showing a histogram of the dot product between the longest and second-longest tracks against the length of the longest track showing clear channel separation. The $^{12}\mathrm{C}(n, n_2)$ is blue, the $^{12}\mathrm{C}(n, \alpha_1)$ is red and the $^{12}\mathrm{C}(n, \alpha_2)$ is green.

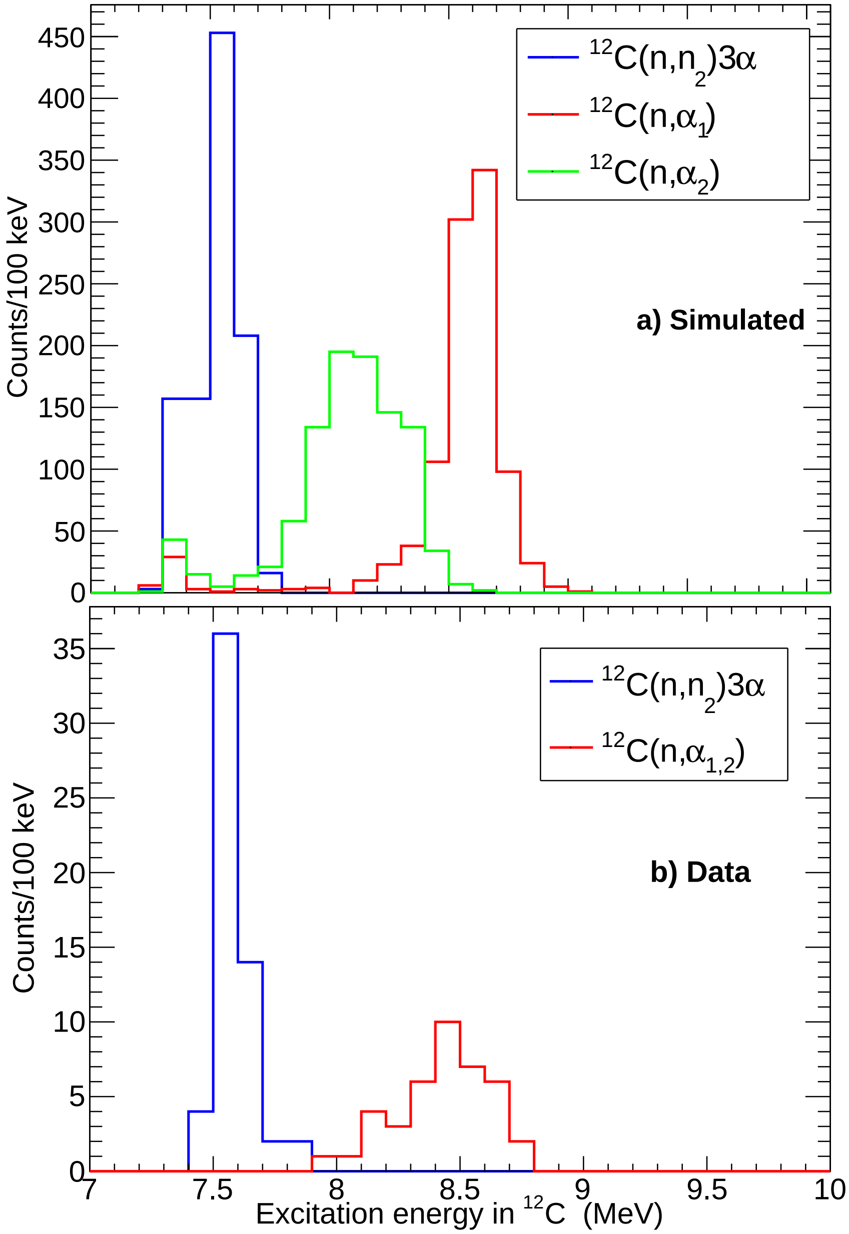

Supplementary Figure 4: Secondary channel separation plot. (a) GEANT4 simulated excitation energies in ^{12}C (at $E_n = 10$ MeV) for the three reaction channels when passed through the analysis process. The separation between the three channels solely by the excitation is clear to see for events where the RANSAC garnered a good fit. (b) Channel separation from data for $E_n = 10$ MeV showing good separation between $^{12}\mathrm{C}(n, n_2)$ in blue and $^{12}\mathrm{C}(n, \alpha_{1,2})$ in red via the excitation energy in ^{12}C reconstructed from the three measured α-particles.

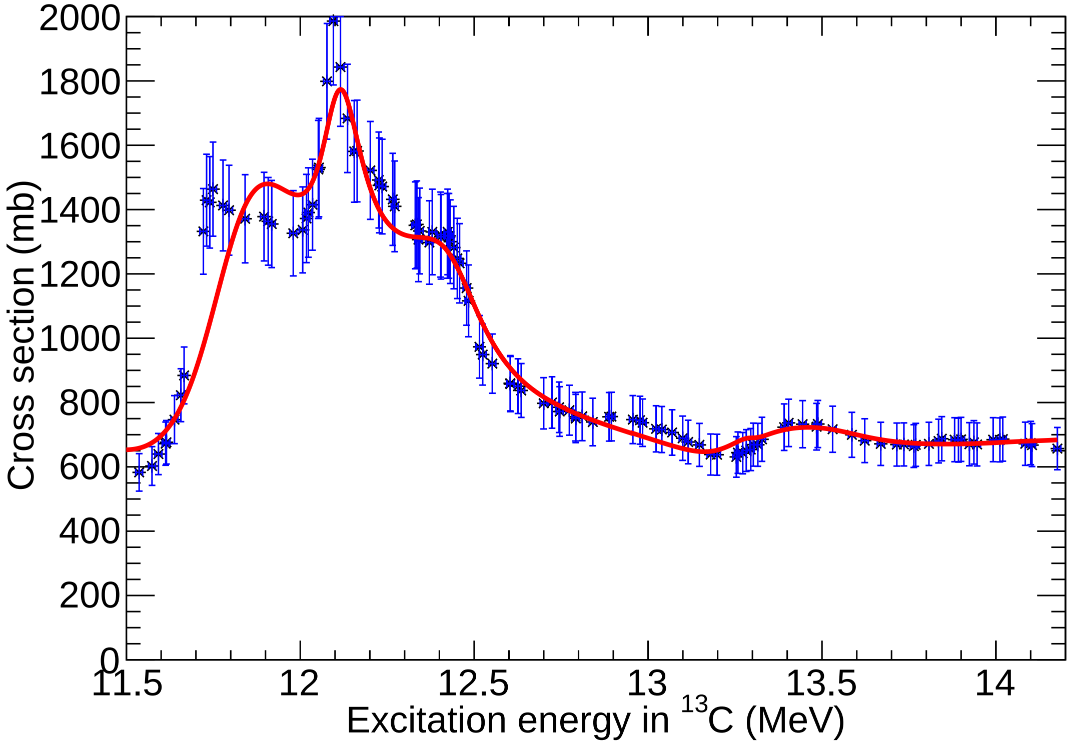

Supplementary Figure 5: R-Matrix fit to data. $^{12}C(n, n_0)$ cross section (points) overlaid with multi-channel R-Matrix fit in red. Error bars show 1 standard deviation.

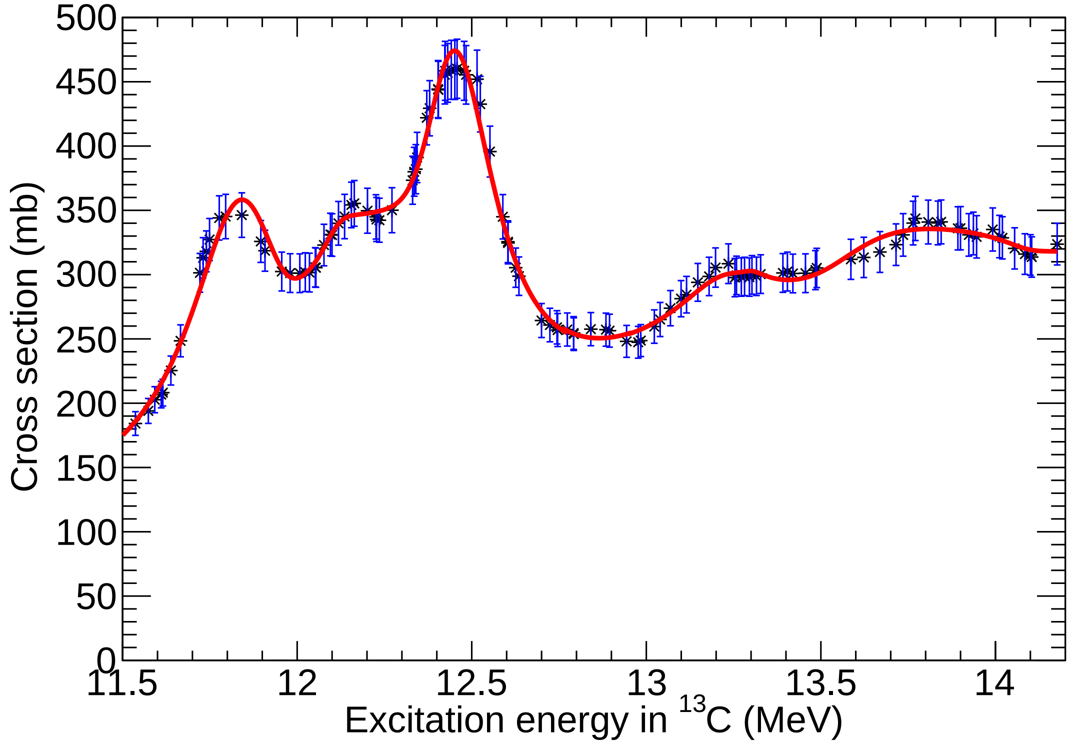

Supplementary Figure 6: R-Matrix fit to data. $^{12}C(n, n_1)$ cross section (points) overlaid with multi-channel R-Matrix fit in red. Error bars show 1 standard deviation.

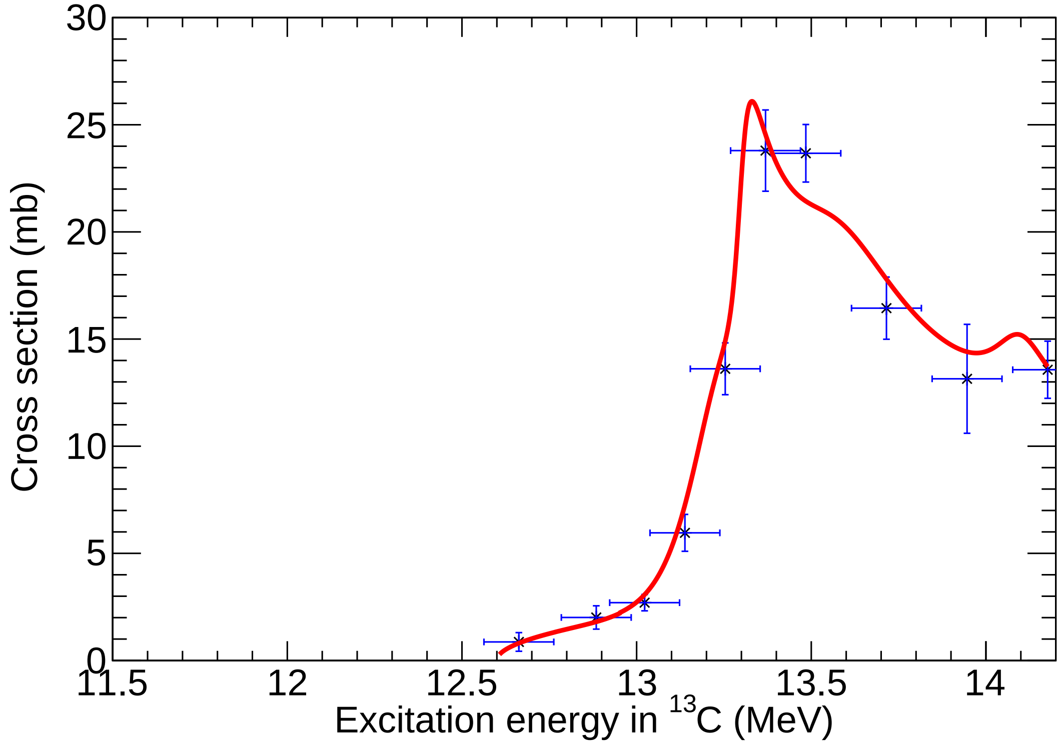

Supplementary Figure 7: R-Matrix fit to data. $^{12}\mathrm{C}(n, n_2)$ cross section from this work (points) overlaid with multi-channel R-Matrix fit in red. Error bars show 1 standard deviation.

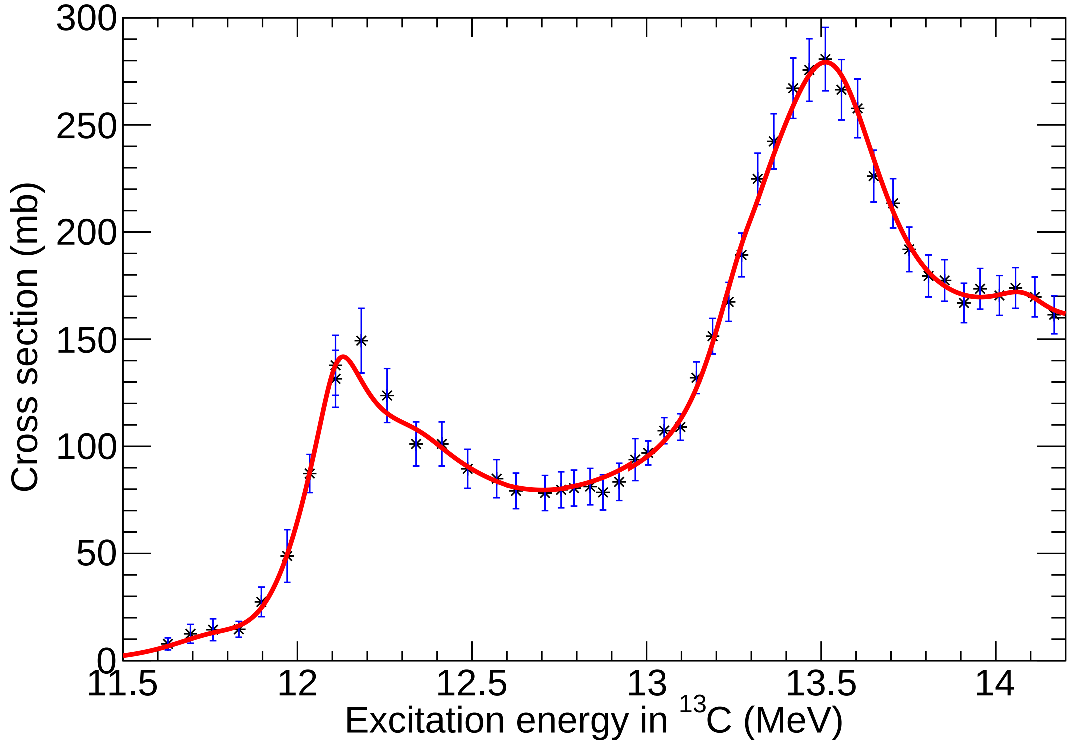

Supplementary Figure 8: R-Matrix fit to data. $^{12}\mathrm{C}(n, \alpha_0)$ cross section (points) overlaid with multi-channel R-Matrix fit in red. Error bars show 1 standard deviation.

Supplementary Table 1: Summary of R-Matrix parameters. Excited states in ^{13}C included in the R-Matrix fit in the astrophysical range of interest

Spin parity	E_x (MeV)	Γ_{n0} (keV)	Γ_{n1} (keV)	Γ_{n2} (keV)	Γ_{α_0} (keV)
$\frac{1}{2}^+$	13.28	0.2	2.7	106.8	15.5
$\frac{3}{2}^-$	13.28	146.5	71.6	44.7	291.3
$\frac{7}{2}^-$	13.57	54	8.1	15.4	366.1
$\frac{5}{2}^+$	13.76	401.9	1065.6	0.7	201.8
